# Anti-*Helicobacter pylori* Biofilm Extracts from *Rubus idaeus* and *Rubus occidentalis*

**DOI:** 10.3390/pharmaceutics16040501

**Published:** 2024-04-05

**Authors:** Rafał Hałasa, Katarzyna Turecka, Urszula Mizerska, Mirosława Krauze-Baranowska

**Affiliations:** 1 Department of Pharmaceutical Microbiology, Medical University of Gdansk, Al. Gen. J. Hallera 107, 80-416 Gdansk, Poland; katarzyna.turecka@gumed.edu.pl; 2 Department of Polymeric Nanomaterials, Centre of Molecular and Macromolecular Studies, Polish Academy of Sciences, ul. Sienkiewicza 112, 90-363 Lodz, Poland; urszula.mizerska@cbmm.lodz.pl; 3 Department of Pharmacognosy, Medical University of Gdansk, Al. Gen. J. Hallera 107, 80-416 Gdansk, Poland; miroslawa.krauze-baranowska@gumed.edu.pl

**Keywords:** antibacterial activity, biofilm, *Helicobacter pylori*, plant extracts, *Rubus* sp., synergism

## Abstract

*Helicobacter pylori* infections are still an important health problem and are directly related to the development of gastric ulcer, gastric adenocarcinoma, mucosal lymphoid tissue lymphoma, and diabetes. At the same time, the number of substances/drugs effective against these bacteria is limited due to increasing resistance. Raw plant materials from various species of the Rubus genus—fruits and shoots—have shown antimicrobial activity in numerous studies against different bacteria, including *H. pylori* in a planktonic form. Research carried out on a model using fragments of intravenous infusions and triphenyl tetrazolium chloride (TTC) as a dye showed that the shoot extract of *Rubus idaeus* ‘Willamette’, the fruit extract of *R. idaeus* ‘Poranna Rosa’, *R. idaeus* and *R. idaeus* ‘Laszka’, and *R. occidentalis* Litacz’ prevent the formation of biofilm by *H. pylori*. Active concentrations inhibiting biofilm formation were 6.65 mg/mL for shoots and 16.65 mg/mL for fruits. However, in the resulting biofilm, the extract from the shoots of *R. idaeus* ‘Willamette’ and the fruit of *R. idaeus* ‘Poranna Rosa’ at a concentration of 16.65 mg/mL was active against living bacteria, and the remaining extracts showed such activity at a concentration of 33.3 mg/mL. In studies on the interaction of the extract with antibiotics on biofilm, the extract from the shoots of *R. idaeus* ‘Willamette’ showed synergy with doxycycline and levofloxacin, additivity with amoxicillin and clarithromycin, and neutrality with metronidazole. *H. pylori* biofilm research was carried out in a newly elaborated research model—culture on fragments of intravenous infusions with the addition of TTC as a marker of living bacterial cells. The research results may constitute the basis for the development of new combination therapies for the treatment of *H. pylori* infections, including its resistant strains. The proposed new biofilm research model, which is cheap and effective, may allow testing of new substances that are potentially more effective against *H. pylori* and other biofilm-forming bacterial strains.

## 1. Introduction

*Helicobacter pylori* is a Gram-negative microaerophilic bacterium [1,2] that causes a number of gastric diseases, including peptic ulcer disease, gastric adenocarcinoma, atrophic gastritis, and mucosa-associated lymphoid tissue lymphoma [3]. It is estimated that approximately 4.4 billion people have *H. pylori* colonizing the gastric mucosa. Three morphological forms of *H. pylori* have been described, namely spiral forms, which are viable and culturable, with strong colonizing ability; coccoid forms, which are viable but not suitable for cultivation (VBNC) and represent persistent forms of this bacterium; and degenerated forms, which are characterized by the breakdown of the cytoplasmic membrane, which is most likely a manifestation of bacterial death [4]. The transition from the spiral to the coccoid form is caused by extreme environmental conditions, including the following: changes in temperature, pH or the presence of antibiotics [5]. There are also reports of the possibility of transformation of *H. pylori* from the coccoid form to the spiral form, stimulated by an environment enriched with growth factors [6,7]

It is commonly believed that *H. pylori* infection in humans is mainly associated with gastritis [8]. Bacteria in the stomach survive due to multiple virulence factors, including urease, vacuolating cytotoxin A (VacA), and spiral shape, and are also suspected to form bacterial biofilms in the stomach [9]. However, various routes of infection are possible. There have been reports that bacteria have been isolated from dental plaque and drinking water sources [8]. Young et al. [10] reported that the spiral and viable coccoid forms of *H. pylori* occur in the oral cavity in the form of a biofilm [2,8]. It has also been proven that bacteria can form biofilms in aquatic systems and can survive from >10 days (spiral form) to 1 year (VBNC coccoidal form) in fresh water [8]. Both places where bacteria occur outside the gastric mucosa are important, both from the point of view of transmitting infections from one person to another, as well as recurrent infections in people undergoing antibiotic therapy [8].

Biofilm is a population of bacteria attached to abiotic and biotic surfaces, surrounded from the outside by a polymeric substance. In the case of *Helicobacter pylori*, biofilm formation begins with a spiral form that attaches to the surface, and then, as a result of cell morphological changes, various forms are created: spiral, rod-shaped, curved, coccoid, and filamentous. Finally, all cells transform into a coconut form that is resistant to environmental factors [11]. Biofilms show tolerance or resistance to antibiotics by expressing efflux pumps (transport proteins that play a role in the export of toxic substances, including antibiotics) [1].

First-line antibiotics for the treatment of *H. pylori* infections are amoxicillin and clarithromycin (may be replaced by levofloxacin), or metronidazole in combination with a proton pump inhibitor. It is recommended to use the drug combination for 10–14 days. In case of failure, second-line therapy is introduced, including the following: proton pump inhibitor, bismuth, metronidazole, and tetracycline [12]; other drugs used include vonoprazan and rifabutin [3]. *H. pylori* antibiotic resistance in Europe currently remains at baseline levels of 21.8% for clarithromycin, 15.8% for levofloxacin, and 38.9% for metronidazole. In the current situation, special attention should be paid to the appropriate level of *H. pylori* eradication and alternative, effective methods of eliminating the pathogen should be proposed [13].

In accordance with WHO recommendations, new compounds active against resistant bacterial strains are being sought. Extracts from raw plant materials serve as rich sources of various biologically active secondary metabolites, including flavonoids, terpenes and saponins, and show potential for discovering compounds with desirable antibacterial properties. Research on raw plant materials, in search of compounds acting against *H. pylori,* has been carried out by many research teams [11,12,14,15,16,17]. There are several reports on the effect of extracts from plants of the *Rubus* genus on *Helicobacter* bacteria. Martini et al. [18] examined the effect of an extract from *Rubus ulmifolius* growing in Italian forests and isolated polyphenols (ellagic acid, gallic acid, quercetin) on *H. pylori*. Park et al. [19] assessed the antibacterial properties of *Rubus crataegifolius* extract. Another study [20] analyzed the effect of the OptiBerry^®^ product containing extracts from six berries, namely raspberries, cranberries, elderberries, strawberries, bilberries, and blueberries on the *H. pylori* ATCC 49503 strain. Goodman et al. [21] compared the antimicrobial activity against *H. pylori PMSS* of commercially available black raspberry (*Rubus occidentalis*), blackberry (*Rubus fruticosus*) and red raspberry (*Rubus idaeus*). In turn, Krauze-Baranowska et al. [22,23] compared the effect of extracts from fruits of three cultivated varieties of *Rubus idaeus* ‘Ljulin’, ‘Veten’, and ‘Poranna Rosa’, one variety of black raspberry—*Rubus occidentalis* ‘Litacz’, and extracts from the shoots of *R. idaeus* ‘Willamette’ on a number of Gram-positive and Gram-negative bacteria, including *H. pylori*. Raw plant materials from species of the *Rubus* genus are a rich source of polyphenolic compounds, among which antimicrobial activity has been demonstrated primarily for sanguiin H-6 from the group of ellagitannins and ellagic acid. So far, the effect of extracts from the above-mentioned raw plant materials on *H. pylori* biofilm has not been investigated.

The interactions of some substances of plant origin with antibiotics used in chemotherapy against *H. pylori* [13] have also been studied, but there are no studies on extracts from *R. ideaus* and *R. occidentalis* in this area.

In our publication, we present the results of research on the influence of selected raspberry fruit and shoot extracts, namely the fruits from *Rubus idaeus* (‘Poranna Rosa’, ‘Laszka’), the fruits from *Rubus occidentalis* ‘Litacz’, and the shoots of *R. idaeus* ‘Willamette’ on the biofilm produced by *H. pylori*. Additionally, a new model for testing *H. pylori* biofilm was developed, using fragments of intravenous infusions and enabling the assessment of the antimicrobial effect of some natural substances, including their interactions with some antibiotics.

## 2. Materials and Methods

### 2.1. Chemical and Reagents

Standard compounds of caffeic acid, ellagic acid, gallic acid, catechin, hyperoside, and isoquercetin were obtained from Fluka (Buchs, Switzerland); procyanidin B_1_, procyanidin B_2_, and quercetin 3-O-glucuronide were obtained from Extrasynthèse (Genay, France); protocatechuic acid, and epicatechin were obtained from Sigma (Steinheim Germany). Sanguiin H-6 was originated from the collection of standards of the Department of Pharmacognosy from Medical University in Wrocław (Poland). All used solvents of analytical and HPLC grade were purchased from Merck (Darmstadt, Germany). Demineralized water was prepared by using Merck Millipore Water Purification System (Burlington, MA, USA).

### 2.2. Strains, Growth Medium, Antibiotics

The *Helicobacter pylori* reference strain ATCC 43504 was used. Brucella agar plates were used to grow bacteria in microaerobic conditions in GENbag microaer, BioMerieux (Marcy-l’Étoile, France). Brucella broth and brain heart infusion broth (BHI) (Becton Dickinson, Franklin Lakes, NJ, USA) supplemented with 5% horse serum was used in antimicrobial tests with tripticasein soy broth (TSB, Biocorp, Lublin, Poland). Antibiotics used in this study include the following: clarithromycin (50 mg/mL, KRKA d.d. Novo mesto Slovenia), metronidazole (5 mg/mL, solution for infusion Polpharma, Starogard Gdanski, Poland) levofloxacin (5 mg/mL, solution for infusion, Pharmathen S.A, Pallini, Greece), ciprofloxacin (10 mg/mL, solution for infusion, KRKA d.d. Novo mesto, Slovenia), doxycycline (20 mg/mL, solution for infusion, Polfa Tarchomin, Warszawa, Poland), and amoxicillin (100 mg/mL, Tarchomin, Warsaw, Poland). A polypropylene infusion set, sterile, non-pyrogenic, and nontoxic (Magromed, Lublin, Poland) was used. For biofilm studies, we used Brucella and BHI broth supplemented with 5% horse serum (GrasoBiotech, Starogard Gdanski, Poland) with or without 0.1% β-cyclodextrin (Merck Life Science Sp. z.o.o., Poznan, Poland). The colorimetric tests for the estimation of metabolic activity of cells, resazurin, MTT, and TTC (Merck Life Science Sp. z.o.o., Poznan, Poland) markers were used. It is used as an oxidation-reduction indicator in cell viability assays for both aerobic and anaerobic respiration. All the tests were incubated at 37 °C. 

### 2.3. Preparation of Dried Extracts from Fruits of R. idaeus and Fruits and Shoots of R. occidentalis

The analyzed fruits and shoots of the varieties of *R. idaeus* (‘Poranna Rosa’, ‘Laszka’, and ‘Willamette’) and *R. occidentalis* (‘Litacz’) were obtained from the collection of the Experimental Fruit-Growing Station of the Research Institute of Pomology and Floriculture in Brzezna (Poland). Two different methods were used to prepare extracts, depending on the type of raw plant material analyzed.

(a) Freeze-dried and powdered fruits (5 g) were extracted in an ultrasonic bath (3 × 10 min) using 30 mL of EtOH:H_2_O (80:20, *v*/*v*). The obtained ethanol–water extracts were filtered and the ethanol was evaporated under reduced pressure. The aqueous residue was lyophilized.
(b) Dried and powdered shoots (10 g) were exhaustively extracted in a Soxhlet apparatus with chloroform and then with methanol. The solvent was removed from the methanol extract under reduced pressure. The resulting dry residue was dissolved in 10 mL of water and lyophilized.

The obtained lyophilizates prior to analysis were dissolved in methanol and filtered through 0.2 µm PTFE syringe filter.

### 2.4. Qualitative and Quantitative HPLC-DAD-ES-IMS Analysis of the Obtained Dried Extracts from Fruits and Shoots of R. idaeus and R. occidentalis 

HPLC–DAD–ESI-MS system (Shimadzu, Japan) was used. Separations were performed on a Kinetex PFP column (100 × 4.6 mm, 2.6 µm) (Phenomenex, Torrance, CA, USA) with the gradient elution program: 0 min–10% B, 60 min–40% B, 65 min–100% B; A: H_2_O/TFA, 100:0.1 (*v*/*v*), B: H_2_O/AcCN/TFA, 50:50:0.1 (*v*/*v*/*v*), at column temp. 20 °C, flow rate 1.0 mL/min, detection UV at λ–254 nm (simple phenols and polyphenols) at at λ–520 nm (anthocyanins). Mass spectra were acquired in negative (NI) ion mode, the nebulizing gas (nitrogen) flow was 1.5 L/min, desolvation line and block temperature were 250 °C and 200 °C, respectively; the remaining experimental parameters used were interface voltage—4.5 kV, the detector voltage—2.0 kV, and the drying gas (nitrogen) flow—16 L/min. The content of ellagic acid and its derivatives, including ellagitannins, was calculated on ellagic acid, and the anthocyanin content was calculated on cyanidin 3-O-glucoside. Moreover, the concentrations of gallic, caffeic, protocatechic acids, epicatechin, and quercetin 3-O-glucuronide were determined in the analyzed extracts.

### 2.5. Broth Microdilution Assay

Microbroth dilution method assay was performed as per the recommendations of the European Committee on Antimicrobial Susceptibility Testing—EUCAST [24]. The dry methanol extracts were dissolved in DH_2_O to a final concentration of 512 µg/mL. After dilution in BHI or Brucella broth with 5% horse serum, the final concentration of the extracts used for the testing of antimicrobial activity ranged from 128 to 0.00625 µg/mL. After incubation, 30 µL 0.2% MTT—and resazurin were added to the samples, and then visual determination of MIC was performed. The tests were performed in at least three repetitions.

### 2.6. Microtiter Biofilm Formation 

The microtiter biofilm formation was described by Chen et al. [25] with modification. *H. pylori* bacteria were grown in BHI or Brucella broth supplemented 5% horse serum, at 37 °C for 72 h with shacking. Totals of 30 µL of bacteria suspensin and 190 µL of broth (BHI or Brucella) were added to 96-well microtiter plates and grown in GENbag microaer without shaking. After 72 h of incubation, 20 µL 0.2% MTT and/or 1% TTC were added and then incubated for another 2 h at 37 °C. The stained biofilm was washed 3 times with PBS and immobilized by adding 0.5% formalin. The biofilm was dissolved (DMSO from MTT, ethanol from TTC), and the OD (OD 550 nm from MTT and OD 495 from TTC) was measured using a microplate reader (Infinite^®^200 PRO, Tecan, Männedorf, Switzerland). The tests were performed in at least three repetitions.

### 2.7. The Effect of Antibiotics on Biofilm Formed on Microtiter

*H. pylori* bacteria were grown in BHI or Brucella broth supplemented with 5% horse serum for 72 h at 37 °C with shaking. Totals of 30 µL of bacterial suspension and 190 µL of broth (BHI or Brucella) were added to 96-well microtiter plates and cultured in a GENbag microaer without shaking After 72 h of incubation, the tested extracts (concentrations 33.3, 16.66, 6.65, and 3.33 mg/mL) and antibiotics (concentrations 512, 256, 128, 64, 32, 16, 8, 4, 2, and 1 µg/mL) were added, and bacteria were grown in a GENbag microaer without shacking. After 72 h of incubation, 20 µL 0.2% MTT was introduced to the studied samples and incubated for 2 h at 37 °C. The stained biofilm was washed 3 times with PBS buffer and immobilized in the 96-well microtiter plates by adding 0.5% formalin. The biofilm was dissolved in DMSO and the OD 550 nm was measured using a microplate reader (Infinite^®^200 PRO, Tecan, Männedorf, Switzerland). The tests were performed in at least three repetitions.

### 2.8. Quantitation of Biofilm Formation on Glass Coverslips

The method was described by Cole et al. [26] with some modification. *H. pylori* biofilm was formed on 24 × 24 glass coverslips in sterile 60 mm-diameter plastic plates in Brucella broth or BHI broth with 5% horse serum. Medium containing 0.1% β-cyclodextrin was incubated in GENbag microaer. According to Bugali et al., [27]. quantitation of bacteria in biofilm was performed using 1% TTC, or 0.2% MTT, and or 0.1% crystal violet. All dyes associated with the biofilms were dissolved in 1 mL of solvent (MTT and crystal violet in ethanol, TTC in DMSO), and 200 µL of the resulting solutions were used to measure absorbance using a microplate reader to determine the amount of biofilm formation. The tests were performed in at least three repetitions.

### 2.9. Biofilm Formation on Fragments of an Intravenous Infusion

The bacteria were grown for 72 h, with shaking, in glass tubes, in 5 mL of broth, under paraffin to limit the access of oxygen. The following broths were used in the studies: TSB, BHI, and Brucella broth with or without 5% sheep blood or 5% horse serum or 0.1% β-cyclodextrin. All tubes contained 2–3 fragments (2 cm) of an intravenous infusion. After 48 h of incubation, 1% TTC was added to glass tubes with either type of culture broth and further incubated for a maximum of 72 h. To another set of cultures, after 72 h, 30 μL of 0.2% MTT was added and incubated at 37 °C for 2–3 h. After incubation, the broth was removed from the tubes and the intravenous infusion fragments were washed 3 times with PBS. Changing the color of the intravenous infusion fragments to purple (MTT) or red (TTC) revealed the formed biofilm. Fragments of the intravenous infusion in the tested broths without bacteria were used as controls All the dye associated with the biofilm was dissolved with 1 mL of DMSO, and 200 µL of the resulting solution was used to measure absorbance using a microplate reader to determine the amount of biofilm formation. The tests were performed in at least three repetitions.

### 2.10. Study of the Effect of Extracts on Biofilm Formation of Intravenous Infusion

*H. pylori* were grown in glass tubes in 5 mL of broth under paraffin, to limit the access to oxygen, with shaking for 72 h. The *H. pylori* grew on the BHI broth supplemented with 5% horse serum. All tubes contained 2–3 fragments (2 cm) of intravenous infusions and antibiotics: doxycycline, levofloxacin (concentration ranging from 128 to 0.5 µg/mL), amoxicillin, clarithromycin, and metronidazole (concentration ranging from 128 to 32 µg/mL) or tested extract from *Rubus* sp. (concentration ranging from 0.5 µg/6.66 to 0.8 mg/mL). After 48 h of incubation, 1% TTC was added to glass tubes with either type of culture broth and further incubated for a maximum of 72 h. To another set of cultures, after 72 h, 30 μL of 0.2% MTT was added and incubated at 37 °C for 2–3 h. After incubation, the broth was removed from the tubes and the intravenous infusion fragments were washed 3 times with PBS. Changing the color of the intravenous infusion fragments to purple (MTT) or red (TTC) revealed the formed biofilm. Fragments of intravenous infusion in the tested broths without bacteria were used as a negative control. The tests were performed in at least three repetitions.

### 2.11. Checkerboard Assays for Planktonic Bacteria

The checkerboard assay method was described by Pillai et al. [28]. BHI broth with the addition of 5% horse serum was used in the research. The antibiotics levofloxacin and doxycycline at concentrations ranging from 0.5 to 0.016 µg/mL and plant extracts at concentrations ranging from 33.3 to 3.33 mg/mL were tested. Bacteria were cultured for 72 h in microaerobic conditions. After incubation, 30 μL of 0.2% MTT and 1% TTC were added to the samples; the results were read using an Infinite 200 PRO Tecan after 2 h of incubation. After incubation, the turbidity of the samples was visually determined and the results were read. The MIC values of the antibiotic and the test extract were determined and substituted into the formula to calculate the Fractional Inhibitory Concentration index (FICI):

FICI = FIC_A_ + FIC_B_ = A/MIC_A_ + B/MIC_B_,

where MIC_A_ and MIC_B_ are the MICs of drugs A (antibiotic) and B alone (plant extract). FIC (index) values were interpreted as follows [29]: FIC index ≤ 0.5 indicates synergism; FIC index > 0.5 to <1.0 shows partial synergism; FIC index = 1.0 indicates addition; FIC index > 1.0 to –4.0 denotes indifference; and FIC index > 4.0 denotes antagonism [30]. The tests were performed in at least three repetitions.

### 2.12. Checkerboard Assays for Biofilm Formation on Fragments of Intravenous Infusion

Bacteria were cultured in glass tubes in 5 mL of broth with shaking under paraffin, in order to limit the access of oxygen, for 72 h. BHI with 5% horse serum broths were used in the studies. All tubes contained 2–3 fragments (2 cm) of intravenous infusion. The following antibiotics were used: amoxicillin, clarithromycin, metronidazole, levofloxacin, and doxycycline at concentrations ranging from 128 to 0.125 µg/mL; the concentration of the plant extract ranged from 33.3 to 0.8 mg/mL. 

After 48 h of incubation, 1% TTC was added to the glass tubes with culture broth and further incubated for a maximum of 72 h. Next, the broth was removed from the tubes and the intravenous infusion fragments were washed 3 times with PBS. A change in the color of the intravenous infusion fragments to red indicated biofilm formation. Fragments of the intravenous infusion in the tested broths without bacteria were used as negative controls. All dye associated with biofilms was dissolved in 1 mL of DMSO and 200 μL of the resulting solution was used to measure absorbance using a microplate reader to determine the amount of biofilm formed. The tests were performed in at least three repetitions.

### 2.13. Scanning Electron Microscope (SEM)

The effect of the plant extracts on the biofilm formed on the surface of intravenous infusion fragments was determined using SEM according to the procedure described by Turecka et al. [31]. A JSM-6010LA scanning electron microscope from JEOL (Akishima, Tokyo, Japan), equipped with an EDX energy dispersive X-ray spectrometer, was used.

### 2.14. Statistical Analysis

All experiments were performed at least 3 times. The intergroup differences were estimated by one- or two-way analysis of variance using Microsoft Excel 2010. All data were additionally analyzed by STATISTICA ANOVA v. 13.3. The distribution of normality of continuous variables was calculated using the Shapiro–Wilk test. The data are presented as mean and standard deviation (±SD). A *p* value was considered as statistically significant when it was less than 0.05.

## 3. Results

### 3.1. Phytochemical Profiles of the Analyzed Dry Extracts from Fruits of R. idaeus and Fruits and Shoots of R. occidentalis

The HPLC-DAD-ESI-MS analysis confirmed the presence of ellagitannins, ellagic acid derivatives, and flavonoids in extracts from *R. occidentalis* ‘Willamette’ shoots and fruits from *R. idaeus* ‘Laszka’, ‘Poranna Rosa’, and *R. occidentalis* ‘Litacz’ (compounds 1–14, 21–40) (Table 1). Anthocyanins were present only in the fruits of *R. idaeus* ‘Laszka’and *R. occidentalis* ‘Litacz’. Identification of compounds was carried out on the basis of comparison with standard compounds and comparison of the obtained chromatographic data (Table 1) with data previously published by us [20,22,32].

**Figure 1:**
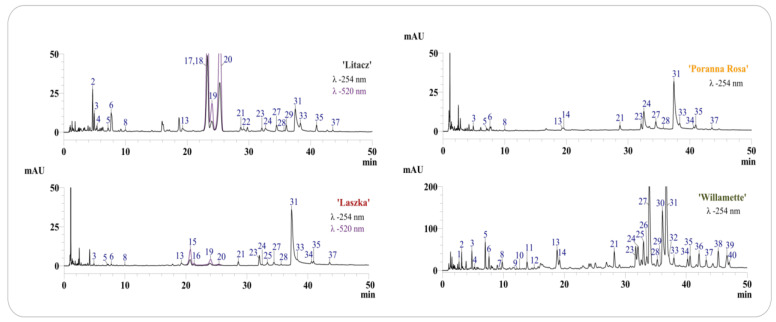
Chromatograms LC-DAD of dry extracts from fruits and shoots of analyzed *R. idaeus* and *R. occidentalis* varieties. Chromatograms with numbering of separated compounds in each variety of *R. idaeus* and *R. occidentalis*, the numbers of peaks correspond to the numbers of identified compounds in Table 1, with detection at λ–254 nm and 520 nm, depending on the variety.

All analyzed fruit extracts contained sanguiin H-6 (31), four isomers of sanguiin H-10 (3, 6, 21, 24), 8 sanguiin H-2 (28), quercetin 3-*O*-glucuronide (33), ellagic acid (27) and its pentoside (23), methylpentoside (35), and acetylpentoside (37). The fruits of the ‘Poranna Rosa’ variety were the only ones to contain epicatechin (14), while the presence of an additional pentoside of ellagic acid (25) was found in the fruits of the ‘Laszka’ variety. Both above-mentioned varieties were also characterized by the presence of an unidentified quercetin pentoside (34). Protocatechuic acid (4) and hyperoside (29) were also detected in the extract from the fruit of the black raspberry ‘Litacz’ (Figure 1, Table 1). The presence of anthocyanins was confirmed in the fruit extracts of *R. idaeus* ‘Laszka’ and *R. occidentalis* ‘Litacz’. Both varieties were characterized by the presence of cyanidin 3-*O*-glucoside (19) and cyanidin 3-*O*-rutinoside (20). Cyanidin 3-*O*-sophoroside (15) and cyanidin 3-*O*-(2^G^-glucosylrutinoside) (16) were detected only in the ‘Laszka’ variety, while cyanidin 3-*O*-(2^G^-xylosylrutinoside) (17), cyanidin 3-*O*-sambubioside (18) (in the form of one peak containing unresolved both compounds), and pelargonidin 3-*O*-rutinoside (22) were only detected in the ‘Litacz’ variety). In addition to the phenolic compounds determined in fruits (excluding anthocyanins), gallic acid (1), chlorogenic acid (9) and caffeic acid (11), procyanidin B_1_ (7) and B_2_ (12), catechin (10), lambertianin C (30), and two additional ellagic acid acetylpentosides (39, 40) were only identified in the shoots of *R. idaeus* ‘Willamette’ (Figure 1, Table 1 and Table 2).

Quantitative determination of ellagitannins, ellagic acid and its derivatives, anthocyanins and selected phenolic acids, epicatechin, and quercetin 3-*O*-glucuronide was carried out in four tested extracts (Table 2). The results of the quantitative analysis confirmed that the extract from the shoots of *R. idaeus* ‘Willamette’ is a many times richer source of polyphenolic compounds compared to the tested fruit extracts. The concentration of ellagic acid in the shoots of *R. idaeus* ‘Willamette’ was approximately 15 times higher than in the tested fruit varieties. The shoots of the ‘Willamette’ variety were also characterized by the highest content of sanguin H-6, the concentration of which was two to five times higher than in the fruit (Table 2). Similarly, the sum of ellagitannins and ellagic acid derivatives in the shoots of the ‘Willamette’ variety were 2–3 times and 4–6 times higher, respectively, compared to the fruits (Table 2).

Quantitative analysis of anthocyanins in the fruit extracts of *R. idaeus* ‘Laszka’ *and R. occidentalis* ‘Litacz’ confirmed that black raspberry fruits are an over 13 times richer source of these compounds (Table 2).

### 3.2. Antimicrobial Activity of Antibiotics and Extracts

The minimum inhibitory concentration (MIC) was defined as the lowest concentration of antibiotic/plant extract inhibiting visible bacterial growth, as read visually or using a microtiter reader after the color change of MTT and resazurin. The obtained MIC values of the antibiotics and extracts tested are shown in Table 3.

The results showed identical MIC values for the tested antibiotics, despite the different media and dyes used in the assay.

Comparing the effectiveness of the two dyes, it was shown that in the remaining samples the weakest signal was provided by Brucella broth with the addition of 5% horse serum and resazurin. In the samples tested, resazurin produces a weaker color signal detecting live bacterial cells than MTT. Based on the results obtained, Brucella broth and MTT dye (3-(4,5-dimethylthiazol-2-yl)-2,5-diphenyltetrazolium bromide) were selected for the next stage of cerebral heart infusion (BHI) studies.

### 3.3. Microtiter Plate Biofilm Formation: Study of the Effect of Extracts on the Produced Biofilm

Detection of *H. pylori* biofilm is mainly limited to checking its level using crystal violet. However, the main goal of research should be to assess the bacteria killed in the biofilm or the prevention of its formation. This study represents the first time (as far as we know), that TTC marker and BHI medium have been used with horse serum to study *H. pylori* biofilm.

**Figure 2:**
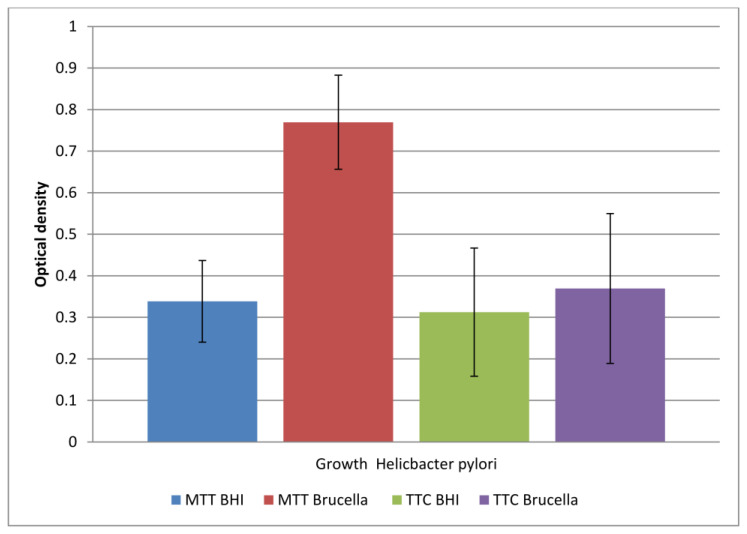
The level of viable cells in a biofilm formed in two different media (BHI and Brucella) labeled with two dyes (TTC and MTT). The results are presented as mean values ± standard deviation (±SD) from three independent experiments. Error bars represent standard deviation. *p* < 0.05 was considered as statistically significant.

The comparison of the obtained results is shown in Figure 2.

The study showed that both media allowed biofilm formation in the wells of the plate within 72 h. Higher detectability of live bacteria present in biofilms was noted with the MTT dye. The OD value corresponding to the biofilm formed in Brucella medium and the selected MTT is twice as large as the other values. This means that in a 96-well titration plate, in the presence of Brucella broth, H. pylori can form a biofilm with more viable cells than in BHI broth. The sample marked in Figure 1 as TTC Brucella gives an OD value two times lower than the sample marked as MTT Brucella; these are samples of the same substrate labeled with a different marker. This means that TTC labels live cells less effectively than MTT on the titration plate. The use of both markers in detecting the amount of live biofilm cells in the BHI broth were at a similar level (sample 1 marked as MTT BHI and sample 3 marked as TTC BHI).

**Figure 3:**
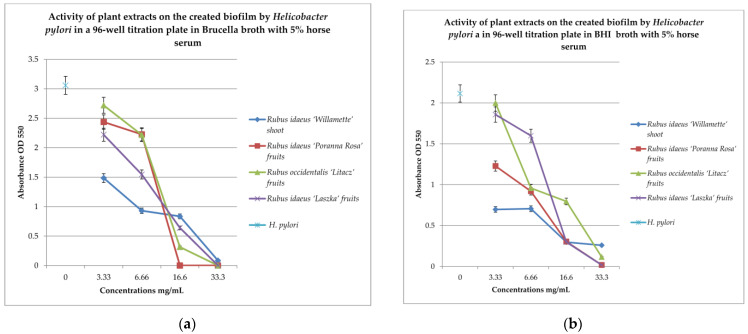
Examination of the activity of plant extracts on the created biofilm by *Helicobacter pylori* in a 96-well titration plate in Brucella (**a**) and BHI media with 5% horse serum (**b**). MTT was used as the marker. Measurements were made at OD _550_. The results are presented as mean values ± standard deviation (±SD) from 3 independent experiments. Error bars represent standard deviation. *p* < 0.05 was considered as statistically significant.

To check the effect of extracts on the created biofilm, BHI and Brucella broth with 5% horse serum was used with MTT as the label. The results are shown in Figure 3a,b.

To check the effect of the extracts on the created biofilm, BHI and Brucella broth with 5% horse serum and MTT were used as the dye. The results are shown in Figure 2a,b. Analyzing the relationship in Figure 2a, it can be noticed that with the increase in the concentration of the extracts examined, the bactericidal properties also increase. The extract from *Rubus idaeus* ‘Willamette’ shoot at the concentration of 3.33 mg/mL turned out to be the most active, killing 50% of the living cells compared to the control. At the same concentration, the extract from *Rubus occidentalis* ‘Litacz’ fruits was found to be the least active, reducing living cells only by 10%. The concentration of 16.6 mg/mL extract from *Rubus idaeus* ‘Willamette’ shoot killed 99% of the *H. pylori* cells, while the remaining extracts show this activity at a concentration twice as high. BHI medium with the addition of 5% horse serum promotes the maintenance of the formed biofilm, but its level is two times lower than in the presence of Brucella medium with the addition of 5% horse serum. At a concentration of 3.33 mg/mL, the most active were the following extracts: *Rubus idaeus* ‘Willamette’ shoot, *Rubus idaeus* ‘Poranna Rosa’ fruits, and *Rubus occidentalis* ‘Litacz’ fruits, causing a reduction in live bacteria of about 50% in relation to the control. On the other hand, about 80% reduction in live bacteria was demonstrated by *R. idaeus* ‘Willamette’ shoot, *R. idaeus* ‘Poranna Rosa’ fruit, and *R. idaeus* ‘Laszka’ fruit, at a concentration of 33.3 mg/mL. Fruit extracts *R. idaeus* ‘Poranna Rosa’ and *Rubus occidentalis* ‘Litacz’, at a concentration of 6.66 mg/mL, decreased the number of viable cells in the biofilm formed by about 50% relative to the control.

### 3.4. Quantitation of Biofilm Formation on Glass Coverslips

The gold standard in *H. pylori* biofilm research is glass coverslip testing using crystal violet, Brucella broth, and β-cyclodextrin. In our research, we wanted to compare the efficiency of biofilm formation using two different substrates along with the detection of live bacterial cells as an indicator of the amount of biofilm formed. Therefore, determination of the amount of biofilm formed on glass coverslips in BHI and Brucella broth, with the addition of 5% horse serum and in the presence or absence of β-cyclodextrin, using three dyes, MTT, CV, and TTC, was performed.

**Figure 4:**
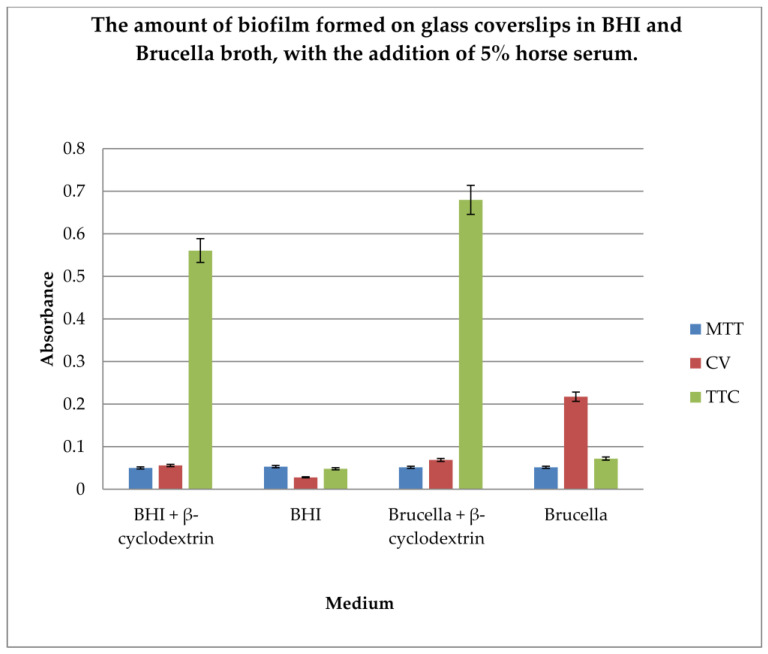
Determination of the amount of biofilm formed on glass coverslips in BHI and Brucella broth, with the addition of 5% horse serum and in the presence or absence of β-cyclodextrin, using three dyes: MTT, CV, and TTC. The results are presented as mean values ± standard deviation (±SD) from three independent experiments. Error bars represent standard deviation. *p* < 0.05 was considered as statistically significant.

The results are shown in Figure 4.

The level of biofilm formation on glass coverslips in BHI and Brucella medium with 5% horse serum, in the presence or absence of β-cyclodextrin, was evaluated. The dyes used to visualize the biofilm level were MTT, crystal violet, and TTC. The biofilm was grown without shaking for 3 days. The highest level of biofilm (with live cells) was obtained when cultured in BHI and Brucella broth with the addition of 5% horse serum and 0.1% TTC-labelled β-cyclodextrin. We have shown that both media with β-cyclodextrin allow the formation of a biofilm, but only TTC showed how many live bacteria can be found there. The TTC level significantly exceeded that achieved by crystal violet.

### 3.5. Biofilm Formation on Fragments of an Intravenous Infusion

**Figure 5:**
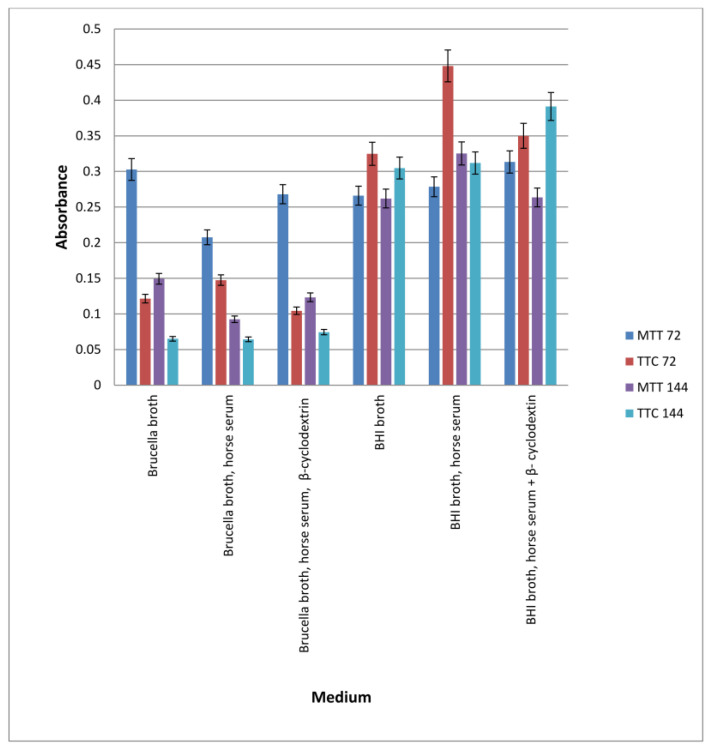
Determination of the amount of biofilm formed on fragments of an intravenous infusion. BHI and Brucella broth, with the addition of 5% horse serum and in the presence or absence of β-cyclodextrin, MTT, and TTC markers, were used. The results are presented as mean values ± standard deviation (±SD) from three independent experiments. Error bars represent standard deviation. *p* < 0.05 was considered as statistically significant.

To demonstrate the possibility of biofilm formation on surfaces made of plastic, experiments in which *H. pylori* bacteria were cultured in the presence of fragments of intravenous infusions in 5 mL broth under paraffin for 72 h with shaking were performed. The BHI and Brucella broth, with or without 5% horse serum or 0.1% β-cyclodextrin, were used. The presence of biofilm was checked after 3 and 6 days. The results are presented in Figure 5.

In all samples tested, the bacteria formed a biofilm. Significantly more biofilm was formed in the presence of BHI than in Brucella broth. Incubation of samples for 3 or 6 days resulted only in significant growth of biofilm (live bacteria) in BHI broth with horse serum and β-cyclodextrin (bacteria were detected by TTC). A large amount of biofilm was also detected in BHI medium containing horse serum, in which the number of viable cells was measured using TTC after 3 days of incubation. Comparing the results presented in Figure 3 and Figure 4, it can be concluded that bacteria form biofilms faster on plastic surfaces than on glass surfaces, in the absence of β-cyclodextrin and serum. Biofilm in BHI broth is detected using MTT and TTC at a similar level, but more live cells are detected in the biofilm after 72 h by TTC.

### 3.6. Study of the Effect of Extracts on Biofilm Formation on Intravenous Infusion

**Figure 6:**
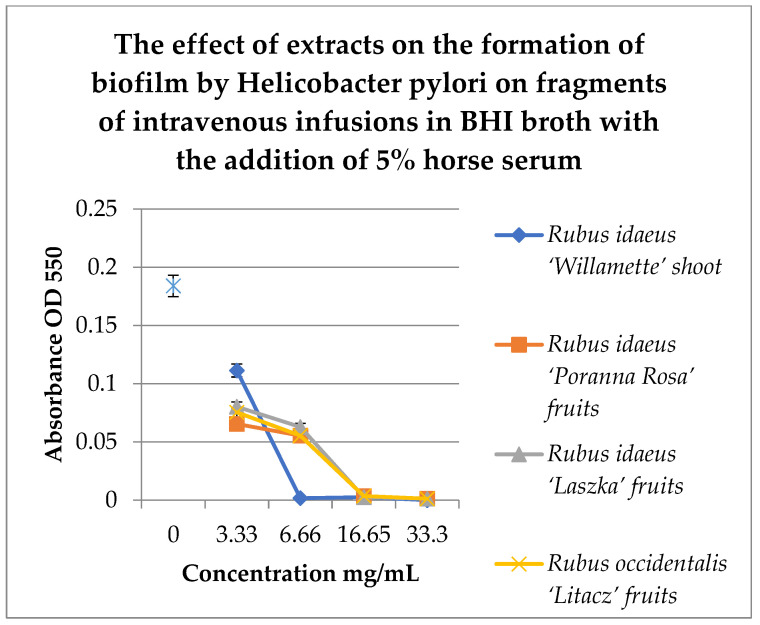
Study of the effect of extracts on the formation of biofilm by *H. pylori* on fragments of intravenous infusions in BHI broth with the addition of 5% horse serum. The determination was carried out by TTC at absorbance OD550. The results are presented as mean values ± standard deviation (±SD) from three independent experiments. Error bars represent standard deviation. *p* < 0.05 was considered as statistically significant.

To study the effect of extracts on the formation of biofilm on fragments of intravenous infusions, BHI broth with the addition of 5% horse serum was used. Samples were incubated in tubes under paraffin at 37 °C for 72 h. MTT was used to detect the biofilm, and the absorbance was measured at 550 nm. The results are shown in Figure 6.

Figure 6 shows that extracts at a concentration of 3.33 mg/mL reduce the amount of living *H. pylori* cells in the formed biofilm. Strong inhibitory properties can be observed at a concentration of 6.66 mg/mL for *Rubus idaeus* ‘Willamette’ shoot, while other extracts from fruits inhibit biofilm formation from a concentration of 16.65 mg/mL.

### 3.7. Study of the Effect of Antibiotics or Extracts on Biofilm Formed in Intravenous Infusions

**Figure 7:**
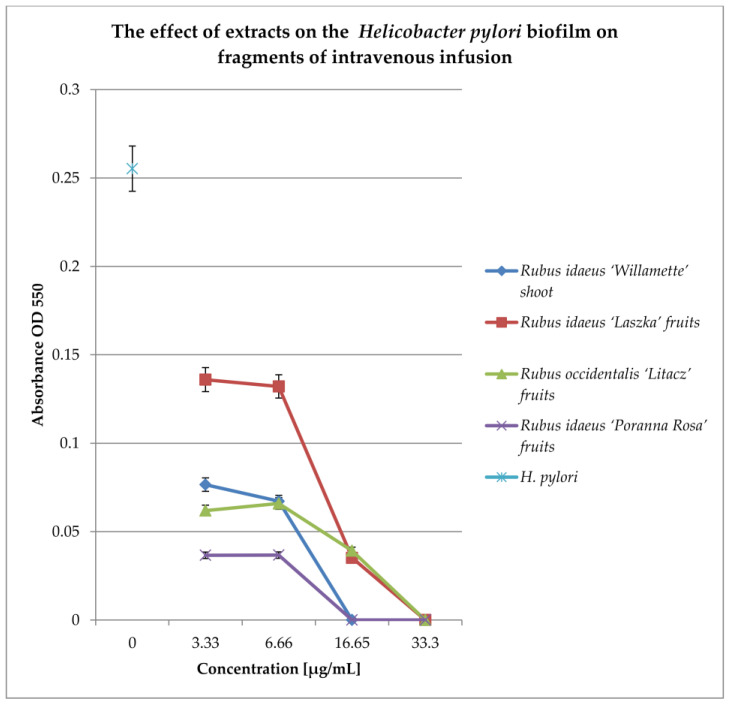
Examination of the effect of extracts on *H. pylori* biofilm formed on fragments of intravenous infusions in BHI broth, including 5% horse serum. The determination was carried out by TTC at OD550. The results are presented as mean values ± standard deviation (±SD) from three independent experiments. Error bars represent standard deviation. *p* < 0.05 was considered as statistically significant.

To check the effect of extracts on the biofilm formed on fragments of intravenous infusions, the technique for research on biofilm formation after 144 h was used. The results are shown in Figure 7.

All extracts at concentrations of 33.3 and 16.65 mg/mL kill most of the bacterial cells in the 144 h biofilm formed by *Helicobacter pylori*. Lower concentrations (6.66 and 3.33 mg/mL) reduce the living bacterial cells in the biofilm by approximately 40%. Only the extract from Rubus idaeus “Laszka” fruits in concentrations of 3.33 and 6.66 causes a reduction in living cells by 20% compared to the control.

### 3.8. Checkerboard Arrays on Planktonic Cells and Biofilm Formation

Nowadays, antibiotic therapy of infection caused by *H. pylori* more often ends in failure due to the bacteria’s resistance to antibiotics. It seems logical to check what interactions occur between antibiotics and the tested extracts. The results for the study of interactions on planktonic bacteria are presented in Table 4.

We used all extracts and only the two most active antibiotics against our *H. pylori* strain (Table 4). All the tested extracts showed synergism with doxycycline, and only the extract of *Rubus occidentalis* ‘Litacz’ showed synergism with levofloxacin. The remaining extracts tested showed indifference with levofloxacin.

To compare the results obtained for planktonic bacteria, we tested the interactions of antibiotics and the extract, as shown in Table 5. We conducted the research on fragments of intravenous infusions.

Among the tested extracts, only the extract from *Rubus idaeus* ‘Willamette’ shoot (the most active) (Table 5) in combination with doxycycline and levofloxacin showed synergy. With amoxicillin it showed additivity, and with clarithromycin and metronidazole, indifference. Similar studies should be carried out with the remaining extracts.

### 3.9. Scanning Electron Microscope (SEM)

**Figure 8:**
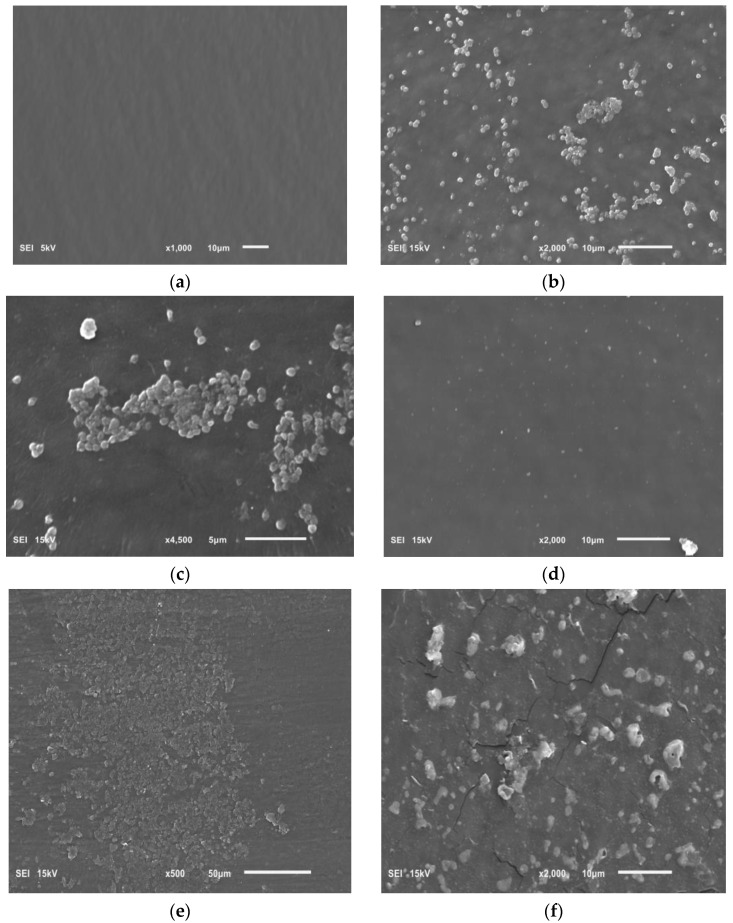
Photo showing the following: (**a**) sterile surface of the intravenous infusion; (**b**,**c**) the biofilm of *H. pylori* on surface of the intravenous infusion; (**d**) the effect of *Rubus idaeus* ‘Willamette’ shoot extract at a concentration of 6.66 mg/mL on forming biofilm; (**e**,**f**) the effect of *Rubus idaeus* ‘Willamette’ shoot extract at a concentration of 6.66 mg/mL on bacteria in formed biofilm.

The effect of the tested extracts on the biofilm formed on intravenous infusion fragments was assessed using SEM. Photos taken using this technique are presented in Figure 8.

Figure 8a shows a clean sterile surface of the intravenous infusion fragment. Figure 8b,c show a *H. pylori* biofilm on the intravenous infusion surface with coccoid/spherical form cells, characteristic of a mature biofilm. Figure 8d shows the surface of an intravenous infusion fragment incubated with a mixture of bacteria and extract (inhibition of biofilm formation).

Figure 8e,f show the remains of bacterial cells exposed to extract from *Rubus idaeus* ‘Willamette’ shoot at a concentration of 6.66 mg/mL. The presence of mature *H. pylori* biofilm visible in the photos (Figure 8b,c) confirms the TTC absorbance (OD 550) of *H. pylori* biofilm control and the presence of live bacterial cells in the biofilm after 72 h, measured using TTC. However, the remains of bacterial cells constituting the biofilm visible in Figure 8d,e are the effect of the extract. The effect shown in Figure 8d,e confirms zero (close to zero) TTC absorbance in samples where the bacteria were killed by the tested extracts, in this case, the extract from *Rubus idaeus* ‘Willamette’ shoot with a concentration of 6.66 mg/mL.

## 4. Discussion

In combating *H. pylori* infections in the human population, it is more important to quickly detect the presence of living bacteria and achieve a bactericidal effect than to achieve only a bacteriostatic effect. This is due to the fact that drug resistance, the ability to form biofilm, and persistent infections caused by *H. pylori* are rapidly increasing. Currently adopted treatment strategies include combined therapy, which involves the use of several antibiotics with different mechanisms of action at the same time. Moreover, the possibility of using a plant extract active against *H. pylori* alongside antibiotics is being sought [19,25,33,34].

Taking into account the fact that the diagnosis of *H. pylori* infection is based only on determining its presence and does not take into account the distinction between living and dead bacterial cells, the aim of the study was to develop a cheaper and simplified method for differentiating the types of *H. pylori* bacterial cells present in the biofilm. We also aimed to assess the impact on *H. pylori* biofilm of plant extracts obtained from the shoots of *Rubus idaeus* ‘Willamette‘ and the fruits *R. idaeus* ‘Poranna Rosa’, *R. idaeus* ‘Laszka’, *R. occidentalis* Litacz, and their combinations with some antibiotics.

The first stage of the research was to confirm the antimicrobial effects of the tested plant extracts against *H. pylori* by determining the concentrations at which they have a bacteriostatic or bactericidal effect against the non-biofilm form of this bacteria. Previously, our research indicated the effect of shoots of *Rubus idaeus* ‘Willamette’ on *H. pylori* and compounds present in fruits and shoots from species of the *Rubus* genus, namely ellagic acid and sanguiin H-6, belonging to the ellagitannins [22,23,32].

The two most common media used for bacterial culture were tested, namely Brucella broth and BHI broth, with the addition of 5% horse serum. Moreover, resazurin and MTT were used as markers to assess the metabolic activity of bacterial cells. Enzymes such as NAD(P)H-dependent cellular oxidoreductases may, under certain conditions, be useful in assessing the number of viable bacterial cells present. The obtained test results were similar for both substrates, but the use of MTT dye gave a stronger marking signal compared to resazurin. The extracts we studied showed inhibitory activity (MIC) against *H. pylori* at a concentration of approximately 7.5 mg/mL. Results of studies on commercially available black raspberries (*Rubus occidentalis*; BRB), blackberries (*Rubus fruticosus*; BB) and red raspberries (*Rubus idaeus*; RRB) against *H. pylori* conducted by Goodeman et al. [21] showed that all berries had antibacterial activity with complete inhibition of *H. pylori* growth at a concentration of approximately 4%. In turn, research on extracts from *Rubus ulmifolius* leaves and isolated individual polyphenols (ellagic acid, gallic acid, quercetin) carried out against *H. pylori* G21 and 10 K strains, showed MBC values after 24 and 48 h of exposure at the level of 1200 µg/mL, 1500 µg/mL and 134 µg/mL, 270 µg/mL, respectively [Martini et al. [18].

The next step in assessing the biological activity of the tested extracts from the *Rubus* genus was to examine their effect on the *H. pylori* biofilm. So far, various research models, substrates and markers have been used to demonstrate the presence of biofilm [9,11,25,26,33,34]. Therefore, in the course of our research, we compared different methods, taking into account different substrates and markers assessing biofilm formation by the *H. pylori* bacterium. Based on the results obtained, we proposed a new model for testing the formation of biofilm with a polypropylene infusion set, which is a model material reflecting the formation of biofilm on various artificial surfaces, including in hospital conditions.

Our studies, including tests in 96-well titration plates in the presence of Brucella broth and BHI as culture media and TTC or MTT dyes, showed that *H. pylori* can form a biofilm with more viable cells on Brucella medium than in BHI broth supplemented with 5% horse serum (Figure 2). Tests of the antibacterial activity of the extracts in terms of biofilm formation by *H. pylori* in a 96-well titration plate showed that the most active was the extract from the shoots of *R. idaeus* ‘Willamette’ at concentrations of 3.33 mg/mL (Brucella medium) and 16.6 mg/mL (BHI broth). The remaining extracts showed lower activity depending on the culture medium used (BHI and Brucella broth with 5% horse serum combined with MTT were used) compared to the control (Figure 3a,b).

Our studies, in which coverslips for biofilm formation by *H. pylori* were used, demonstrated that the highest level of live cells in biofilm is obtained in terms of BHI and Brucella broth with 5% horse serum culture and with addition of 0.1% TTC-labelled β-cyclodextrin. The TTC level significantly exceeds that achieved by crystal violet. Triphenyl tetrazolium chloride (2,3,5-triphenyl-2H-tetrazolium chloride) (TTC) is a redox indicator commonly used in biochemical experiments. The white compound is enzymatically reduced to red TPF (1,3,5-triphenylformazan) in living cells due to the activity of various dehydrogenases (enzymes important in the oxidation of organic compounds and thus cellular metabolism).

Studies showing that *H. pylori* can form biofilms in vitro on plastics (titer plates, tubes with treated water, on tissues) allow us to assume that bacterial cells in contact with plastics such as stents in vivo, prostheses, or central inserts, may behave similarly. An additional element contributing to the creation of these formations is the presence of tissue fluids, including serum and the nutrients it contains. The use of a microcapillary flow system [35] allows for in vitro biofilm research, which largely simulates the possibilities of biofilm formation in, e.g., water pipes and flow cannulas.

This prompted us to propose a new research model for *H. pylori* biofilms on fragments of intravenous infusions. Studies on biofilm formation on the mentioned above elements showed that the highest number of living bacteria in the biofilm, incubated for 3 days in BHI broth with horse serum and β-cyclodextrin, were detected using TTC indicator. Biofilm in BHI broth was detected using MTT and TTC at a similar level. However, more live cells were detected in the biofilm after 72 h by TTC.

The possibility of *H. pylori* biofilm formation on intravenous infusions, detected with TTC dye, was confirmed by scanning electron microscope (SEM) images (Figure 8b,c). The photos show coccidal/spherical *H. pylori* forms. This form results in physiological changes in the bacteria, including tolerance to variable environmental factors. Transformation may lead to the development of a viable but non-culturable (VBNC) phenotype by spherical *H. pylori* forms, but still metabolically active [11]. Thus, our research method makes it possible to see these forms, check whether they are alive, and at the same time, test the activity of compounds on this form.

The rapid increase in *H. pylori* resistance to conventional treatment methods and the formation of a biofilm structure on biotic and abiotic surfaces forces various groups of researchers to look for quick and reliable methods for assessing the impact of tested compounds on *H. pylori* cells. This is important, particularly since the current commonly used methods are long-lasting, expensive, and difficult to read/interpret, and maintaining *H. pylori* culture is also difficult. Recently, one of the methods, which was used to assess the impact of fruit preparations from the *Rubus* genus [21] on the planktonic form *H.pylori* was the method described as high-throughput metabolic microarray assay

Most of the mentioned methods require the purchase of ready-made reagents and appropriate equipment for measurements and data processing, which increases the costs of tests. The time needed to perform them is very similar to the method developed and presented in this manuscript. Our research fits into the general trends prevailing in research on *H. pylori* and includes not only testing the effect of plant extracts on *H. pylori* planktonic cells, but also research on the formation of biofilm and their impact on the emerging biofilm. The proposed method is cheap, based on available and inexpensive materials, and to read the results, only a reader of titration plates measuring absorbance is needed.

Our research on the influence of tested extracts from *Rubus* sp. on the formation of biofilm showed, that concentrations of 6.66 mg/mL and 16.65 mg/mL of extracts from the shoots and fruits of *R. idaeus*, respectively, inhibit the formation of biofilm on fragments of the intravenous infusion. However, it should be emphasized that all extracts at concentrations of 33.3 mg/mL and 16.65 mg/mL reduced the number of viable bacteria in the 144 h biofilm formed by *H. pylori*. In relation to these results, we assessed the effect of extracts from *Rubus* sp. fruits and shoots not only on the formation of biofilm by *H. pylori* but also on the already formed biofilm. In SEM photos, the prevention of the formation of biofilm was observed (Figure 8d) and the destructive effect of the extract from *R. idaeus* ‘Willamette’ shoots on the biofilm was clearly visible (broken cells, Figure 8e,f). The above results also confirmed that the use of TTC dye for biofilm research allows for a clear distinction between living and dead cells. At the same time, we showed that the model we tested can be used to conduct research on the activity of plant matrices with different chemical compositions.

The performed studies on potential interactions of extracts from *Rubus* sp. and the two most active antibiotics against planktonic bacteria *H. pylori* showed synergism of all extracts with doxycycline, and synergism with levofloxacin only of the extract from *R. occidentalis* ‘Litacz’. In turn, studies on the interaction of antibiotics and the extract of *R. idaeus* ‘Willamette’ shoots carried out on fragments of intravenous infusions showed synergism with doxycycline and levofloxacin, additivity with amoxicillin, and neutrality with clarithromycin and metronidazole.

Krzyżek et al. [9] studied the interaction of myricetin (MYR) with the antibiotics amoxicillin, clarithromycin, tetracycline, metronidazole, and levofloxacin. They used the checkerboard test, the LIVE/DEAD kit, and used also fluorescence microscopy to assess potential synergistic effects. Their research results showed that 1/4 × MIC of MYR is able to decrease the MICs of all tested antibiotics by 4–16 times (FICI = 0.31–0.5). The 1/8 × MIC of MYR also increased the activity of all tested antibiotics (the interactions were additive, FICI = 0.625). The 1/16 × MIC of MYR additively increased only the activity of amoxicillin, metronidazole, and levofloxacin (FICI = 0.56), but not clarithromycin and tetracycline (FICI = 2.0).

The combination of RF and UL at a concentration of 75 µg/mL each showed complete inhibition of *H. pylori* colonization. All 11 clinical *H. pylori* strains and 2 reference strains used were tested for synergistic effects of RF and UL with amoxicillin, clarithromycin, and omeprazole and showed a similar inhibitory effect on *H. pylori* colony formation. It was found that the strong anti-*H. pylori* properties of *R. crataegifolius* extract are related to the presence of ellagic acid (14.2 mg/g dry extract) and catechin (30.5 mg/g dry extract).

In the presented studies, the extract of *R. idaeus* ‘Willamette’ shoots turned out to be more active in preventing biofilm formation on fragments of intravenous infusion compared to other fruit extracts. Taking into account the results of our chromatographic analyses of the tested extracts from *Rubus* sp. And data from the literature [22,23], it is possible that its activity against *H. pylori* is related not only to the presence of ellagitannins, including sanguiin H-6 and the ellagic acid and its derivatives [present in the highest concentrations compared to another tested extracts, 598.5 mg/100 g d.w. (sum of ellagitannins content), 455.6 mg/100 g d.w., and 40.7 mg/100 g d.w., respectively] but also epicatechin (1538.2 mg/100 g d.w.), quercetin 3-*O*-glucuronide (166.9 mg/100 g d.w.), gallic acid (20.7 mg/100 g d.w.), and caffeic acid (74.9 mg/100 g d.w.), absent or present but in significantly lower amounts in other extracts. However, in relation to the biofilm formed on the fragments of the intravenous infusion, the extracts that did not contain anthocyanins had the best inhibitory effect on its growth, i.e., the fruit extract of *R. idaeus* ‘Poranna Rosa’ and the extract of the shoots of *R. idaeus* ‘Willamette’. Their biofilm-degrading effect was comparable, although the content of sanguin H-6 and ellagic acid and its derivatives was lower by approximately two- and six-fold, respectively, compared to the extract from *R. idaeus* “Willamette” shoots.

In our previous studies [22,23], we also demonstrated a stronger growth inhibitory effect against the planktonic form of *H. pylori* of the extract from the shoots of *R. idaeus* ‘Willamette’, compared to extracts from fruits of different cultivars of *R. idaeus* (‘Ljulin’,’Veten’, and ‘Poranna Rosa’) and *R. occidentalis* (’Litacz’). It was suggested that it is associated with a higher content of sanguiin H-6 and free ellagic acid. It is believed that ellagic acid, by binding to the bacterial membrane, may destabilize its structure, thus disturbing the functionality of the bacterial cell. More detailed studies on the mechanism of antimicrobial activity, especially against *H. pylori*, ellagitannins of the *Rubus* genus and ellagic acid and its derivatives, would certainly be interesting.

Martini et al. [18] examined the extract of *R. ulmifolius* and the compounds isolated from it—ellagic acid, gallic acid, rutin (quercetin-3-rutinoside), and kaempferol—as effective agents against *H. pylori*. Rutin (quercetin-3-rutinoside) is known to have antimicrobial activity against some Gram-positive and Gram-negative bacteria, and ellagic acid kills *H. pylori* by inhibiting the activity of arylamine N-acetyltransferase. Kaempferol causes a significant decrease in the number of *H. pylori* cells in the stomachs of gerbils after oral administration. It was suggested that the mechanism of antibacterial action of *R. ulmifolius* extract and its polyphenols may be related to the inactivation of bacterial ion pumps by affecting enzymes that regulate the flow of copper and other metal cations through membranes. The activity of the extract after 24 h of exposure was weaker than the activity of individual compounds, which can be explained by the lower concentration of individual compounds in the extract and the presence of other constituents that do not have antibacterial activity. When discussing the antimicrobial effect of ellagitannins and ellagic acid derivatives, it should be taken into account that these compounds are decomposed in the gastrointestinal tract first to ellagic acid and then, under the influence of intestinal microflora, to urolithins, compounds with virtually no antimicrobial activity [20]. Therefore, their antimicrobial effect will be important when used topically, when applied to the skin, or the mucous membranes of the mouth or throat.

Krauze-Baranowska et al. [22] proposed the use of raspberry extracts (from fruits and/or shoots), among others, in the form of lozenges, in order to prevent or limit infections of the respiratory and digestive tract caused by various pathogenic bacteria or viruses. The ability to eat raspberries as components of our daily diet, thanks to wide, almost year-round access to these fruits is a certain health benefit. It should also be emphasized, that some compounds from raspberry fruits and shoots identified as antimicrobially active may constitute starting compounds for the synthesis of derivatives with much greater antibacterial properties. The demonstrated synergy of *Rubus* sp. fruit extracts with doxycycline and the shoots of *R. idaeus* ‘Willamette’ with doxycycline and levofloxacin, as well as the revealed additivity of amoxicillin, may constitute the basis for conducting clinical trials assessing the effectiveness of combined therapy of the above extracts with antibiotics for the treatment of *H. pylori* infections. Our test results showed also that *H. pylori* may form a biofilm on medical materials used in various therapeutic procedures, including on stents or in water treatment systems used, e.g., for artificial respiration in hospitals where the presence of *H. pylori* has been detected. The effect of inhibiting the formation of *H. pylori* biofilm on fragments of intravenous infusions in the presence of various extracts from Rubus sp. has been revealed. In the future, it can be used to protect various hospital surfaces by covering them with a layer of natural substances.

## 5. Conclusions

In our experiments, we have shown that the best medium for testing *H. pylori* biofilm in 96-well titration plates is the Brucella broth medium, in which the metabolic activity of cells is detected by MTT. Among the tested extracts from fruits and shoots of various species of the *Rubus* genus, the most active against *H. pylori* were extracts from the fruits of *R. idaeus* ‘Poranna Rosa’ and the shoots of *R. idaeus* ‘Willamette’, which did not contain anthocyanins. We considered the best medium for testing *H. pylori* biofilm on fragments of intravenous infusions to be BHI medium supplemented with 5% bovine serum, which was characterized by the highest percentage of live bacteria after 72 h of incubation, detected using the TTC marker. The developed conditions were considered model for the study of *H. pylori* biofilm. In this model, we showed that the extracts from the fruits of *R. idaeus* ‘Poranna Rosa’ and the shoots of *R. idaeus* ‘Willamette’ are the most active in inhibiting the emerging *H. pylori* biofilm, while the extract from the shoots of *R. idaeus* ‘Willamette’ showed activity against bacteria in formed by *H. pylori* in the biofilm. The results of interaction studies with planktonic bacteria H. pylori showed that all tested extracts from Rubus sp. had a synergistic effect with doxycycline, and only the Rubus occidentalis ‘Litacz’ extract showed synergism with levofloxacin. However, studies on the interaction of antibiotics and extracts of Rubus idaeus ‘Willamette’ shoots performed on fragments of intravenous infusions showed synergism with doxycycline and levofloxacin. The test results were confirmed using SEM microscopy.

## Figures and Tables

**Table 1:** Chromatographic data of polyphenols and phenolic acids identified by LC-DAD/ESI-MS in the shoots of *R. idaeus* ‘Willamette’ and the fruits of *R. idaeus* ‘Laszka’, ‘Poranna Rosa’, and *R. occidentalis* ‘Litacz’.

	Peak Number/Compound	t_R_ (min)	UV (nm)	ESI-MS (*m*/*z*) [M – H]^−^/[M – H + TFA]^−^
1	Gallic acid *	2.7	214, 270	169^−^/283^−^
2	Unknown compound	3.2	314	577^−^
3	Sanguin H-10 isomer	4.8	250, 270	301^−^, 783^−^, 1567^−^/897^−^
4	Protocatechic acid *	5.3	260, 287	153^−^/267^−^
5	Unknown compound	7.0	304, 329	341^−^/455^−^
6	Sanguiin H-10 isomer	7.6	250, 270	301^−^, 783^−^, 1567^−^/897^−^
7	Procyanidin B_1_ *	9.6	278	577^−^/691^−^
8	Unknown compound	9.8	250, 270	617^−^, 633^−^/747^−^
9	Chlorogenic acid *	12.3	235, 300sh, 324	353^−^/467^−^
10	Catechin *	12.7	278	289^−^/403^−^
11	Caffeic acid *	13.9	235, 296sh, 320	179^−^/293^−^
12	Procyanidin B_2_ *	15.1	278	577^−^/691^−^
13	Unknown compound	18.8	250, 270	1567^−^, 1718^−^
14	Epicatechin *	19.2	278	289^−^/403^−^
15	Cyanidin 3-*O*-sophoroside *	20.7	278, 515	611^+^
16	Cyanidin 3-*O*-(2^G^glucosylrutinoside) *	21.3	282, 514	757^+^
17	Cyanidin 3-*O*-(2^G^xylosylrutinoside)	23.3	279, 518	727^+^
18	Cyanidin 3-*O*-sambubioside *	23.3	279, 516	581^+^
19	Cyanidin 3-*O*-glucoside *	24.0	279, 514	449^+^
20	Cyanidin 3-*O*-rutinoside *	25.3	277, 520	595^+^
21	Sanguin H-10 isomer	28.2	251, 270	301^−^, 783^−^, 1567^−^/897^−^
23	Ellagic acid pentoside	31.7	251, 359	433^−^
24	Sanguiin H-10 isomer	32.1	250, 270	301^−^, 783^−^, 1567^−^
25	Ellagic acid pentoside	33.0	252, 359	433^−^
26	Unknown compound	33.5	250, 270	1250^−^, 1251^−^
27	Ellagic acid *	33.9	252, 366	301^−^/415^−^
28	Sanguiin H-2	35.2	251, 270	1103^−^
29	Hyperoside *	35.8	258, 348	463^−^/577^−^
30	Lambertianin C	36.1	251, 270	1401^−^
31	Sanguiin H-6 *	36.7	250, 270	934^−^, 935^−^, 1869^−^, 1870^−^
32	Isoquercetin *	37.1	256, 262sh, 353	463^−^/577^−^
33	Quercetin 3-*O*-glucuronide *	38.0	254, 352	477^−^/591^−^
34	Quercetin pentoside	40.2	263, 351	433^−^
35	Ellagic acid methylpentoside	40.6	252, 357	447^−^/561^−^
36	Unknown compound	42.1	230, 273	447^−^, 935^−^, 1083^−^
37	Ellagic acid acetylpentoside	43.3	250, 353	475^−^/589^−^
38	Unknown compound	45.2	230, 273	1117^−^/1231^−^
39	Ellagic acid acetylpentoside	46.7	251, 359	475^−^/589^−^
40	Ellagic acid acetylpentoside	47.1	251, 350	475^−^/589^−^

* standard compound.

**Table 2:** Content of phenolic compounds in dry extracts from fruits and shoots of *R. idaeus* and *R. occidentalis* varieties [mg/100 g d. w.].

Chemical Compound	Fruits *R. id.* ‘PR’	Fruits *R. id.* ‘Lasz’	Fruits *R. occ.* ‘Lit’	Shoots *R. id* ‘Will’
Ellagic acid	32.1	24.6	29.6	455.6
Ellagic acid derivatives				
Ellagic acid pentoside	26.8	56.4	23.5	195.1
Ellagic acid methylpentoside ^1^	24.5	22.9	27.9	51.6
Ellagic acid acetylpentoside ^1^	21	22.7	20.6	174.0
sum of contents	72.3	102.0	72.0	420.7
Ellagitannins				
sanguiin H-2 ^1^	19.9	20.0	19.7	44.3
sanguiin H-6 ^1^	140.5	189.0	82.9	339.5
sangwin H-10 isomer ^1^	132.8	84.3	116.1	183.6
lambertianin C ^1^	–	–	–	31.1
sum of contents	293.2	293.3	218.7	598.5
Anthocyanins				
Cyanidin 3-*O*-glucoside	–	56.4	253.1	–
Cyanidin 3-*O*-rutinoside ^2^	–	14.0	1195.0	–
Cyanidin 3-*O*-sophoroside ^2^	–	122.1	–	–
Cyanidin 3-*O*-(2^G^-glucosylrutinoside) ^2^	–	16.1	–	–
Cyanidin 3-*O*-(2^G^-xylosylrutinoside)^2^	–	–	1312.0 *	–
Cyanidin 3-*O*-sambubioside ^2^				
Pelargonidin 3-*O*-rutinoside ^2^	–	–	37.4	–
sum of contents	–	208.6	2797.5	–
Others—phenolic acids, flavonoids				
Gallic acid	–	–	–	20.7
Caffeic acid	–	–	–	74.9
Protocatechic acid	–	–	15.4	20.6
Epicatechin	65.5	–	–	1538.2
Quercetin 3-*O*-glucuronide	15.7	10.1	74.4	166.9

Abbreviations: *R. id.* ‘PR’—*R. idaeus* ‘Poranna Rosa’, *R. id.* ‘Lasz’—*R. idaeus* ‘Laszka’, *R. occ.*’Lit’—*R. occidentalis* ‘Litacz’, *R. id.* ‘Will’—*R. idaeus* ‘Willamette’; ^1^ calculated on ellagic acid; ^2^ calculated on cyanidin 3-*O*-glucoside; * given as a sum due to separation as a single peak.

**Table 3:** MIC values of antibiotics and *Rubus sp*. extracts [mg/mL].

Antibiotics	MIC	
	BHI Broth with 5% Horses Serum	Brucella Broth with 5% Horses Serum
Amoxicillin	0.0160 ± 0.01	0.016 ± 0.01
clarithromycin	>0.128	>0.128
doxycycline	<0.000625	<0.000625
levofloxacin	0.000125 ± 0.01	0.000125 ± 0.01
metronidazole	>0.128	>0.128
Extracts		
*Rubus occidentalis* ‘Litacz’ fruits	7.8 ± 0.50	7.8 ± 0.50
*Rubus idaeus* ‘Laszka’ fruits	7.5 ± 0.50	7.5 ± 0.50
*Rubus idaeus* ‘Poranna Rosa’ fruits	7.2 ± 0.50	7.2 ± 0.50
*Rubus idaeus* ‘Willamette’ shoot	7.4 ± 0.50	7.4 ± 0.50
Ellagic acid	0.125 ± 0.05	0.125 ± 0.05

The results are presented as mean values ± standard deviation (±SD).

**Table 4:** Interactions of *Rubus* spp. extracts with antibiotics on planktonic cells.

Antibiotics	MIC Antibiotic Alone [µg/mL]	MIC Antibiotic Combination [µg/mL]	Extracts	MIC Extracts Alone [mg/mL]	MIC Extracts Combination [mg/mL]	FICI	Outcome
Levofloxacin	0.5	0.016	*Rubus idaeus* ‘Willamette’ shoot	33.3	33.3	1.032	Indifference
Doxycycline	0.125	0.016			3.33	0.228	Synergy
Levofloxacin	0.5	0.016	*Rubus idaeus* ‘Poranna Rosa’ fruits	16.65	16.65	1.032	Indifference
Doxycycline	0.125	0.016			3.33	0.328	Synergy
Levofloxacin	0.5	0.016	*Rubus occidentalis* ‘Litacz’ fruits	33.3	16.65	0.532	Synergy
Doxycycline	0.0625	0.016			3.33	0.356	Synergy
Levofloxacin	0.25	0.016	*Rubus idaeus* ‘Laszka’ fruits	33.3	33.3	1.064	Indifference
Doxycycline	0.125	0.016			3.33	0.228	Synergy

**Table 5:** Interactions of *Rubus idaeus* ‘Willamette’ shoot extracts with antibiotics on biofilm formed in intravenous infusions.

	MIC Antibiotic Alone [µg/mL]	MIC Antibiotic Combination [µg/mL]	MIC *Rubus idaeus* ‘Willamette’ Shoot [mg/mL]	MIC Extracts Combination [mg/mL]	FICI	Outcome
Amoxicillin	128	32	6.66	3.33	1	Addition
Clarithromycin	128	128		6.66	2	Indifference
Metronidazole	128	128		3.33	1.5	Indifference
Levofloxacin	0.5	0.25		0.8	0.62	Synergy
Doxycycline	0.25	0.125		0.8	0.62	Synergy

## Data Availability

Data are contained within the article.
